# Improving the Brain Delivery of Chemotherapeutic Drugs in Childhood Brain Tumors

**DOI:** 10.3390/cancers11060824

**Published:** 2019-06-13

**Authors:** Silvia Triarico, Palma Maurizi, Stefano Mastrangelo, Giorgio Attinà, Michele Antonio Capozza, Antonio Ruggiero

**Affiliations:** Pediatric Oncology Unit, Fondazione Policlinico Universitario A. Gemelli IRCCS, Università Cattolica Sacro Cuore, 00168 Rome, Italy; silviatriarico@libero.it (S.T.); palma.maurizi@unicatt.it (P.M.); stefano_mastrangelo@yahoo.it (S.M.); gioatt@hotmail.com (G.A.); micheleantoniocapozza@gmail.com (M.A.C.)

**Keywords:** cerebrospinal fluid (CSF), blood-brain barrier (BBB), intrathecal (IT) chemotherapy, lumbar puncture (LP), Ommaya reservoir, personalized medicine

## Abstract

The central nervous system (CNS) may be considered as a sanctuary site, protected from systemic chemotherapy by the meninges, the cerebrospinal fluid (CSF) and the blood-brain barrier (BBB). Consequently, parenchymal and CSF exposure of most antineoplastic agents following intravenous (IV) administration is lower than systemic exposure. In this review, we describe the different strategies developed to improve delivery of antineoplastic agents into the brain in primary and metastatic CNS tumors. We observed that several methods, such as BBB disruption (BBBD), intra-arterial (IA) and intracavitary chemotherapy, are not routinely used because of their invasiveness and potentially serious adverse effects. Conversely, intrathecal (IT) chemotherapy has been safely and widely practiced in the treatment of pediatric primary and metastatic tumors, replacing the neurotoxic cranial irradiation for the treatment of childhood lymphoma and acute lymphoblastic leukemia (ALL). IT chemotherapy may be achieved through lumbar puncture (LP) or across the Ommaya intraventricular reservoir, which are both described in this review. Additionally, we overviewed pharmacokinetics and toxic aspects of the main IT antineoplastic drugs employed for primary or metastatic childhood CNS tumors (such as methotrexate, cytosine arabinoside, hydrocortisone), with a concise focus on new and less used IT antineoplastic agents.

## 1. Introduction

Childhood cancers include many malignancies that also occur in adults, such as acute lymphoblastic leukemia (ALL) which is by far the most common, followed by central nervous system (CNS) tumors, lymphomas and certain bone cancers (osteosarcoma and Ewing sarcoma). Cancers that are exclusive to children include neuroblastoma, Wilms tumor, rhabdomyosarcoma, and retinoblastoma. Improvements in the treatment of the childhood cancers have positively influenced overall disease-free survival, such as in ALL [1]. “CNS prophylaxis” with repeated cycles of intrathecal (IT) methotrexate (MTX) has replaced CNS prophylactic irradiation in children with low risk ALL, drastically reducing the incidence of CNS relapse from 50% to 23% [2,3].

“CNS prophylaxis” for pediatric ALL derived from the recognition of CNS as a sanctuary site protected from the systemic chemotherapy by the meninges, the cerebrospinal fluid (CSF) and the blood-brain barrier (BBB) [4,5]. 

For most antineoplastic agents, the total CSF exposure following administration of a systemic dose is less than 10% of the systemic exposure, as shown in Table 1. Consequently, IV administered chemotherapy is usually not very efficacious in primary and metastatic CNS tumors. [6].

In this review, we summarize the principal aspects of physical and biochemical protection of the brain, describing the several alternative strategies experimented during time to improve brain delivery of chemotherapy in primary or metastatic childhood CNS tumors (low and high- grade glioma, medulloblastoma, ependymoma, metastatic ALL and lymphoma), such as BBB disruption, intra-arterial (IA), intracavitary and IT chemotherapy. Moreover, we provided an overview of pharmacokinetics and toxic aspects of IT antineoplastic agents commonly used in pediatric oncology for primary or metastatic CNS tumors.

## 2. Physical Protection of the Brain: Meninges and CSF

The human brain is soft and physically protected by bones of the skull, the meninges and the CSF. The meninges present three layers in children and adults: the dura mater, the arachnoid and the pia mater [7]. The dura mater is tough, strong and composed of fibrous connective tissue. The arachnoid is a middle meninx that forms a loose brain covering; below the arachnoid there is a wide subarachnoid space filled with CSF and large blood vessels. The pia mater is a deep meninx composed of delicate connective tissue on the brain surface [8]. Usually considered as protective membranes, the meninges play a prominent role in the development and maintenance of the CNS. They differentiate at the third month of intrauterine life, playing a role in ontogenesis, by inducing proliferation and differentiation of neuroblasts and axonal growth. Moreover, they are in constant evolution from their formation to senescence.

The CSF is a clear liquid, similar in composition to blood plasma, without blood cells and with less protein and different ion concentration than plasma. In fact, CSF is composed of water for 99%, compared to the 92% of plasma. It is lower than plasma in protein, K^+^ and urea, but is higher in Na^+^, Mg^++^ and Cl^−^ concentration. The mean CSF volume is 150 mL in adults, with 25 mL in the ventricles and 125 mL in subarachnoid spaces, but with marked interindividual variations [9]. Gur et al. reported that CSF volume is 164.5 ± 47.8 mL in adults, with 31.9 ± 17.8 mL in the ventricles, 0.95 ± 0.62 in the third ventricle and 132.6 ± 43.2 mL in extraventricular spaces [10]. 

CFS serves two purposes: physical protection of the neuraxis and a chemical role in the homeostasis of cerebral interstitial fluid and neuronal environment. The buoyancy of the CSF reduces the weight of the brain, preventing the brain from crushing under its weight. It forms a liquid cushion that assures hydro-mechanical protection of the CNS from blows and trauma. CSF also plays a prominent role in brain development, by regulation of the brain interstitial fluid homeostasis, the electrolyte balance, the circulation of active molecules and elimination of catabolites [11].

CSF secretion in adult varies from 400 to 600 mL/day, at approximately 25 mL/h. It is predominantly, but not exclusively, secreted by the choroid plexuses inside the ventricles, under neuroendocrine and hormonal modulation. A minimal poorly defined role in the CSF secretion is also played by the extrachoroidal secretion, derived from the ependymal epithelium, from the extracellular fluid and the cerebral capillaries across the blood-brain barrier [12,13]. 

CSF circulates over the ventricles, with a pulsatile and unidirectional flow along subarachnoid spaces through three foramina: the lateral ventricles, the third and fourth ventricles and the subarachnoid space. From the fourth ventricle, it enters the subarachnoid space between the arachnoid and pia mater. CSF is reabsorbed mainly into venous blood across cranial and spinal arachnoid villi and minimally via paraneural sheaths of nerves into lymphatics. CSF is renewed four to five times per 24 h in young adults. The reduction of the CSF turnover rate during ageing leads to accumulation of catabolites in the brain and CSF observed in certain neurodegenerative diseases. CSF pressure varies according to the age, from 3-4 mmHg in infants to 10–15 mmHg in adults, depending from a dynamic equilibrium between CSF secretion and absorption, influenced by systolic pulse wave, respiratory cycle, abdominal pressure, jugular venous pressure, state of arousal, physical effort and posture [14]. The analysis of cerebrospinal fluid through lumbar puncture (LP) procedures can provide information on diagnosis and it may be therapeutic in certain conditions [15]. 

## 3. Biochemical Protection of the Brain: Blood Brain Barrier (BBB)

The brain is the best-perfused organ in the body, with over 100 billion capillaries, a total length of 400 miles and a total surface area of 20 m^2^. The vasculature of central nervous system supplies the brain of nutrients and oxygen, but the presence of a protective BBB between the blood compartment and the brain is an essential prerequisite to assure correct neuronal functioning of the brain [16].

The BBB develops during fetal life, but it is not yet entirely formed and shows unique properties not present in the adult type [17]. Still, recent data have showed that intercellular tight junctions and many of the influx and efflux transporters form already since embryonal and fetal life in the interfaces between blood, brain and CSF [18,19]. However, these barrier mechanisms are more fragile with a major susceptibility of the developing brain to drugs, toxins and pathological conditions (such as inflammation, hypoxia, hyperbilirubinemia, exposure to environmental toxicants). Consequently, the exposure to pharmacologically molecules or toxins during fetal and neonatal life may impair neuronal division, migration, differentiation and synaptogenesis [20]. 

In the developed brain the areas of the brain without BBB are known as “circumventricular organs”, including pineal body, subfornical organ, median eminence, neurohypophysis, area postrema, vascular organ of the lamina terminalis [21]. 

The BBB provides a protective mechanism that helps to maintain a stable environment for the brain, by highly selective regulation of the exchange of ions, nutrients, metabolites and potentially toxic substances between blood and the brain [22]. 

**Figure 1:**
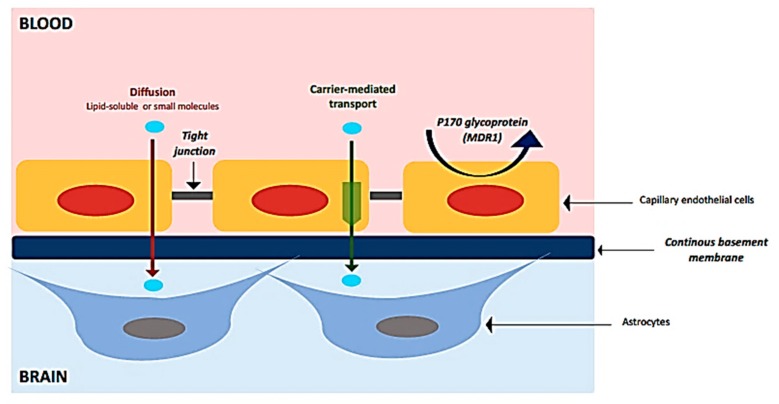
Main characteristics of blood-brain barrier (BBB).

The BBB is made by the endothelial cells of the brain capillaries, that are in continuous interactions with astrocytes, pericytes and perivascular macrophages, forming the “neurovascular unit” (NVU) [23]. In brain capillaries, the endothelial cells are closely connected each other by tight junctions that fuse brain capillary endothelia together into a continuous cellular layer (Figure 1). The tight junctions restrict the paracellular diffusion between the endothelial cells to ions and other polar solutes, blocking penetration of macromolecules. In fact, only great lipid and non-polar solutes may passively diffuse through the cell membrane and cross the endothelium. Conversely, the presence of solute carriers (SLCs) allows the transport of many essential polar molecules, such as glucose, amino-acids and nucleosides, into the CNS. Moreover, receptor-mediated transcytosis (RMT) can transport macromolecules such as peptides and proteins, across the cerebral endothelium; positively charged macromolecules may be transported across the endothelium through adsorptive-mediated transcytosis (AMT) [24]. 

The passage of drugs through the BBB is influenced by several conditions: molecular weight (large molecules do not easily pass through the BBB), ionization at physiological pH (molecules that have a high electrical charge are slowed), liposolubility (low lipid-soluble molecules do not simply penetrate into the brain) and protein binding [25]. 

Furthermore, the achievement of therapeutic concentration of drugs in CNS is complicated by the presence of ATP-Binding Cassette (ABC) efflux transporters, a distinct set of efflux protein localized on the BBB. These efflux transporters are neuroprotective, because they limit the brain entry of neurotoxins; however, they could also restrict the entry of many therapeutics, contributing to CNS pharmacoresistance [26,27].

In humans, P-gp is the best known multidrug resistance (MDR) transmembrane protein, involved in ATP-dependent drug efflux pump, extruding several potentially toxic substances such as various anticancer drugs [28,29]. In addition to P-gp, BCRP is also consider to have a major role in in the control of molecular traffic across the endothelial cells and in drug efflux at the BBB [30]. Recently, in their in vivo study Blakeley et al. have confirmed the direct involvement of BCRP in MTX transport across the BBB, showing that the extrusion of MTX is significantly reduced in BCRP knockout model compared with the wild type [31,32]. Both P-gp and BRCP are expressed early during embryonal life; moreover, while P-gp level increases widely from the perinatal to adult stage, BCRP expression remains stable during development [33,34]. 

Numerous CNS pathologies (such as stroke, trauma, infectious or inflammatory processes, HIV, epilepsy, pain, lysosomal storage disease, brain tumors) involve BBB dysfunction, which can range from mild and transient to chronic barrier breakdown [35]. 

The structure of tumor blood vessels in aggressive brain tumors (such as glioblastoma multiform) is very different from normal blood vessels, including a high endothelial proliferation with widespread infiltration of surrounding tissue. The loss of tight junction in the tumor endothelium leads to the disruption of BBB in the primary tumor site, but not in the infiltrative tumor areas, that are consequently the most difficult lesions to treat. Treatment of primary tumors may be facilitated by the permeable vessels, but not in the infiltrative tumor areas, were the BBB is provided of tight junctions [36,37]. 

Besides, the tumor vascular system maintains the other biological components of the BBB, such as multidrug resistance-associated proteins. Consequently, many anti-cancer drugs are large, hydrophobic and unable to easily go through the BBB, but they are also substrates for the MRD efflux pumps also expressed by tumor vessels [38]. 

## 4. Failed Approaches

Over the years, various strategies have been developed to improve drug delivery into the brain, bypassing the BBB [39]. Although they have failed in their purpose, as they are often not suitable for the pediatric age due to their invasiveness, we describe them in order to address future investigations.

### 4.1. BBB Disruption (BBBD): Osmotic and Bradykinin Receptor-Mediated BBBD

In 1972 Rapaport et al. suggested for the first time that intra-arterial administration of hyperosmotic solutions (such as mannitol which is the most widely used, but also arabinose, lactamide, saline, urea, several radiographic contrast agents) may produce temporary shrinkage of endothelial cells, with consequently opening of tight junctions for several hours, allowing in this time window the delivery of chemotherapeutical agents [40]. 

BBBD seems to be temporary and reversible. Moreover, the non-selective opening of the BBB allows an uncontrolled influx of low and high molecules (such as albumin and plasma proteins) into the brain, with a high risk of seizures and reversible aphasia or hemiparesis [41].

The clinical benefit of osmotic BBBD has not been established for the treatment of less sensitive tumors like gliomas, whereas it seems to increase survival in patients affected by primary lymphoma and other chemo-sensitive tumors [42]. In their study Doolittle et al. established that intra-arterial chemotherapy with an osmotic opening of the BBB results in a high degree of tumor response in patients with chemotherapy-sensitive tumors (such as PNET, germ cell tumor and cancer metastasis) [43]. 

Osmotic BBBD has a more limited practice in children than in adult. In 2006 Hall et al. conducted a study among eight patients (median age 11 years) with diffuse pontine glioma (DPG), treated with monthly osmotic BBBD chemotherapy with a low toxicity profile and survival times longer than those previously reported in other DPG series, concluding that further examination of this treatment for pediatric patients with DPG or other malignant brain tumors was necessary [44].

An alternative attempt to osmotic BBBD is based on the use of an intra-carotid arterial infusion of bradykinin which stimulates receptors on the BBB, inducing opening of tight junctions via second messengers [45]. RMP-7 (cereport or lobradimil) is a bradykinin analogue with improved systemic exposure, designed to induce the bradychinin B_2_ receptors expressed on the endothelial cells. Lobradimil increases permeability of the BBB within minutes following the infusion, by separating the tight junctions between the endothelial cells of the brain capillaries, with the restoration of BBB within 20 to 60 min from the end of the infusion [46,47]. Lobradimil may be used in addition with a chemotherapeutic agent like carboplatin, allowing an enhanced uptake of carboplatin into and near the tumor area [48]. Warren et al. studied the combination of lobradimil and carboplatin in pediatric patients with primary brain tumors in a phase II trial. Still, they demonstrate that the combination of lobradimil and carboplatin is not able to improve the response rate and the time to disease progression in childhood high-grade gliomas and brainstem gliomas [49]. Thus, future studies should further define the biologic conditions in which this drug actually works and the chemical properties of the drugs that enter into the brain because of lobradimil. 

### 4.2. Intra-Arterial Chemotherapy (IA)

IA chemotherapy is a form of regional delivery to brain tumors, designed to enhance the intra-tumor concentrations of a given drug, in comparison with the IV route. The rationale of IA chemotherapy consists of obtaining high local concentrations of a drug with a steep dose-antitumor activity curve. In IA infusion chemotherapy is administered through a catheter which is inserted into the femoral artery and ends in the carotid artery. This procedure may imply an increased risk of periorbital pain, visual loss (up to 10% with carmustine), hearing loss (up to 15% with cisplatin), seizures, confusion and neurocognitive deficits. The risk of these toxicity seems to be related to the heterogeneous distribution of the drug through the various branches of the carotid artery, while the incidence of neurological side effects is significantly improved since the use of novel selective micro-catheters [50].

IA delivery of chemotherapy is well-established for the treatment of hepatocellular carcinoma and retinoblastoma [51,52]. Over the years, several preclinical and clinical studies have demonstrated that IA chemotherapy may have a potential therapeutic benefit for low- and high-grade gliomas and cerebral lymphomas. 

Not all drugs have the appropriate metabolism and pharmacokinetic profile for IA usage. The R_a_ (Regional advantage) equation defines the pharmacologic advantage that a drug may have when administered IA versus the IV route:

R_a_ = 1 + [Cl_tb_ × (total body clearance)/F (blood flow)]

R_a_ is maximized by a rapid total body clearance of the infused drug, which allows, after the “first pass” (during the venous recirculation phase), to extract by the tumor tissue a relative smaller amount of drug than the amount extracted during the “first pass” [53]. The drugs with the most appropriate Ra for IA chemotherapy (ranked in descending order) are carmustine and other nitrosoureas, cisplatin, carboplatin, etoposide and methotrexate [54]. However, a survival benefit for IA drug delivery, in comparison with IV administration, has not been proven in phase III trials and further clinical studies are required to determine the appropriate role for IA chemotherapy in the treatment of primary brain tumors, especially in children [55]. 

### 4.3. Intracavitary Chemotherapy 

Another strategy for circumventing the BBB is the intracavitary chemotherapy (IC), which consists in the implantation of a biodegradable polymer wafer, chemotherapy-impregnated, into the brain or tumor cavity at the time of surgery. This procedure allows obviating these problems, providing the local sustained release of chemotherapeutic agents to the tumor site, prolonging local exposure with minimal systemic toxicity. Gliadel wafers are biodegradable polymer loaded with BCNU (carmustine), which has considerable systemic toxicity and a short half-life in serum [56]. Gliadel wafers are FDA-approved treatment of new-diagnosed and recurrent adult glioblastoma. Their efficacy and safety have been demonstrated in randomized controlled trials which showed that this approach may delay both clinical and radiological progression, increasing the survival rate [57,58,59]. Moreover, their use is currently controversial among neurosurgeons, due to the increased risk of complications, such has seizures, edema, infection, CSF leaks, obstructive hydrocephalus (due to dislodgment of the wafer) [60,61,62]. 

In their study, Sardi et al. reported the cases of three pediatric patients with recurrent anaplastic brain ependymoma, first treated with surgery, then followed by of intracavitary BCNU wafers (Gliadel) implantation in combination with low-doses oral etoposide. Their approach was not effective for the treatment of refractory anaplastic ependymoma. Further studies are necessary to define the potential role of BCNU wafer implantation in the tumor bed after the first tumor resection in addition to consolidated systemic chemotherapy in children with high-risk brain tumors [63].

## 5. Invasive Drug Delivery Directly in the CSF: Intrathecal Chemotherapy (IT)

The administration of antineoplastic drugs directly in the CSF allows to bypass the selective filter of BBB, achieving significant concentrations of the antineoplastic agents in CSF, while reducing the likelihood of systemic toxicity [64]. In fact, at relatively smaller doses, the CSF drug concentrations are more consistent with the IT route, for the smaller CSF volume than the blood compartment (140 vs. 3500 mL), for longer half-lives in CSF than in plasma and lastly for the almost negligible CSF drug clearance by metabolic inactivation and/or protein binding [65]. IT drug delivery can be performed through two methods: into the lumbar thecal sac by LP (intralumbar injection) or into lateral ventricles trough a subcutaneous reservoir and ventricular catheter, called Ommaya reservoir (intraventricular injection) [66].

### 5.1. Intraventricular Injection

The Ommaya reservoir is a subcutaneous device, with a catheter inserted in one of the lateral ventricles of the brain, providing direct access to ventricular CSF [67]. Drugs are injected with a syringe in the ventricular reservoir, after having extracted a CSF volume comparable to the volume of the administered drug and followed by cleaning with about 2–4 mL of 0.9% sodium chloride solution [68]. 

This technique is indicated for patients that required prolonged treatments and when LP could be difficult to perform. Additionally, the advantage of this approach is a more homogeneous drug distribution and a higher drug concentration in the subarachnoid space compared with LP. Better clinical results are achieved especially for the treatment of CNS leukemia or of neoplastic meningitis due to solid tumors [69]. Conversely, it seems to have limited effectiveness for parenchymal masses, because the concentrations of most drugs fall to insignificant levels at few millimeters from the ependymal surface [70]. 

Moreover, the implantation of Ommaya reservoirs may cause long-term complications, including catheter obstruction, hemorrhage and infections. Peyrl et al. analyzed their 20-year experience with Ommaya reservoirs in 98 children with brain tumors, concluding that complications may be reduced with specific training to all workers involved in implanting and accessing the device, with careful attention to strict aseptic conditions [71]. Gerber et al. in their retrospective analysis of 31 consecutive pediatric patients with Ommaya reservoirs, found infection as the most frequent complication. They concluded that further prospective studies are demanded to evaluate preventive measures, such as the administration of peri-operative antibiotics and the use of an antimicrobial coating of catheters [72]. 

### 5.2. Intralumbar Injection

LP is an invasive technique commonly used in pediatric oncology as a diagnostic procedure, but also therapeutically, to inject medications directly into the subarachnoid space [73]. Patients can be in lateral decubitus or sitting positions, which is the preferred position for infants and children [74]. The lowest necessary CSF volume, usually 5–8 mL is extracted for analyses in children and adolescents, 2–3 mL in newborn and infants. Besides, when LP is conducted as therapeutically procedure, it is recommended to slowly administrate the drug, approximately over 3 to 5 min, to reduce the risk of subsequent headaches. Before IT administration it’s necessary to extract a CSF volume equivalent to the volume of chemotherapy instilled; different authors have described injection of volumes ranging from 5 mL to 15 mL [75].

Repeated LP are stressful and painful; therefore, pharmacological sedation is recommended in pediatric patients. Maurizi et al. conducted 252 lumbar punctures under deep sedation with propofol and ketamine in 25 children with ALL, achieving satisfactory sedation and analgesia care in all patients, reducing the risk of traumatic LP and improving comfort and quality of life (QoL) [76]. 

After drug injection, it is recommended to stay at least 1 h supine, flat on the back on a 15–30 degree incline with the feet elevated above the head (Trendelenburg position), to facilitate the penetration of the drug in the brain ventricles, avoiding its prolonged presence in the spinal cord and consequently its absorption through the venous vertebral plexus into the blood. In fact, the slow and unidirectional CSF flow limits the distribution of the drug, which results in relatively low and variable concentration in the ventricular CSF. Studies in animals have shown that ventricular levels of MTX at 1 h after intralumbar injection were as much as 1000 higher in animals kept prone or in Trendelenburg position, compared with CSF concentration in animals kept upright [77]. 

LP may determine several complications, such as post-puncture headache, lower back pain, nerve root irritation, infections or hemorrhages. Another disadvantage consists in the potential injection or leaking of the drug into the subdural or epidural space, which seems to occur in about 10% of LP. Additionally, delayed clearance of intrathecal chemotherapy may be observed in a lot of conditions that interfere with CSF flow. In contrast, the patients with a ventriculo-peritoneal or ventriculo-atrial shunt are at increased risk of a rapid clearance of the drug from the CSF space by the shunt, with an inadequate drug exposure [78].

### 5.3. Pharmacokinetics and Toxicity of Standard Agents for IT Administration 

Despite the presence of numerous systemically active drugs, only a few chemotherapeutic agents are currently available for IT delivery in pediatric oncology, such as MTX, cytosine arabinoside (Ara-C), corticosteroids, and thiotepa. More recently, some other antineoplastic agents are under evaluation for IT administration. In Table 2 we summarize the chemical structures, relevant properties and clinical uses of these pharmacological agents.

There are several antineoplastic drugs limited to systemic administration, such as melphalan, chlorambucil, cisplatin, mitoxantrone, dactinomycin, mercaptopurine and vincristine [79]. Their IT administration is extremely dangerous and usually life-threatening. In particular, vincristine is very neurotoxic and if administered IT produces quickly and fatal ascending leptomeningitis and ventriculitis, which require prompt CSF lavages to remove the drug [80,81,82].

**Figure 2:**
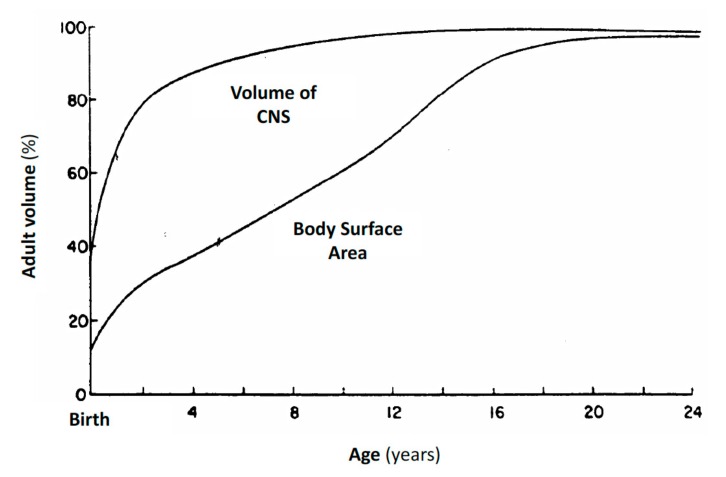
CSF volume compared to BSA in infants and young children. The CSF volume increases at a more rapid rate than BSA, reaching adult volume after the first three years of age (adapted from Bleyer et al. [83]).

As shown in Figure 2, CSF volume is age-related in infants and young children, because it increases more rapidly than body surface area (BSA), reaching adult levels after the first three years of age. Thus, IT chemotherapy in children is usually administered with age-related dosage and not based upon BSA, to obtain an increased therapeutical effect, reducing neurotoxicity [83]. In Table 3 age-related dosage of triple intrathecal (TIT) chemotherapy used in pediatric oncology are reported [84].

#### 5.3.1. MTX

IT MTX has been used for over 50 years in pediatric oncology for the prevention and treatment of CNS blast cell infiltration in acute leukemias and lymphomas, either as a single agent or in combination with Ara-C and hydrocortisone, in the so called “triple intrathecal chemotherapy” [85]. 

The pharmacokinetics of IT MTX is different from systemic administration. Elimination of MTX from the CSF is biphasic, with an initial elimination half-life (t_1/2_) of 4.5 h and a final t_1/2_ of 14 h. MTX clearance is provided by CSF reabsorption, thus it mainly depends from CSF flow. In fact, MTX slowly diffuses from CSF to the plasma compartment, therefore closely repeated IT infusions may behave as a prolonged IV infusions, causing systemic toxicity [86]. 

Although, after intralumbar infusion, CSF concentration of MTX is about 100 times higher than plasma, the CSF ventricular concentration reaches approximately only the 10% of the simultaneous lumbar CSF concentration. Numerous studies warned that intraventricular administration of MTX may provide higher and prolonged ventricular CSF concentration. Ventricular Ommaya reservoir facilitates drug administration, giving repeated small intrathecal doses of MTX over an extended period (for example 1 mg every 12 h for 6 doses). This “concentration times time (C × T) approach” increases the duration of exposure of tumor cells to antineoplastic drug and avoids excessive peak concentrations, simultaneously increasing the potential efficacy and decreasing toxicity [87,88]. 

The risk of neurotoxicity from IT MTX is augmented in the presence of obstructive hydrocephalus, meningeal leukemia or leptomeningeal spread of solid tumors which may interfere with MTX CSF clearance [89]. Potential MTX-neurotoxicity is generally categorized into three forms: acute, sub-acute, chronic. The most frequent form is the acute one, which occurs several hours to few days after MTX IT injection, potentially influenced both by dose and frequency of IT exposure. Acute toxicity presents as a chemical aracnoiditis, with meningism, headache, nuchal rigidity, back pain, fever, and CSF pleocytosis [90]. 

Rarely patients may develop a sub-acute toxicity within 2 or 3 weeks of MTX IT injection, probably due to a persistent toxic drug concentration in the CSF, with the onset of reversible or irreversible paraplegia, myelopathy or encephalopathy, characterized by limb weakness, ataxia, cranial nerve palsies, visual impairment, seizures or coma [91]. 

A late and chronic neurotoxicity may occur months or even years after MTX IT treatment, presenting with progressive demyelating leukoencephalopathy, with limb spasticity, dementia or coma. The addiction of cranio-spinal irradiation or the simultaneous repeated administration of high-dose IV MTX seem to enhance the risk of chronic MTX neurotoxicity [92].

Finally, an accidental MTX IT overdose is associated with an acute life-threatening toxicity, which requires immediate rescue. Different approaches may be useful if promptly applied, such as CSF drainage, ventricololumbar perfusion, systemic administration of high-dose leucovorin and steroids, intrathecal instillations of carboxypeptidase-G_2_, which hydrolyzes MTX in an inactive metabolite [93,94,95,96].

#### 5.3.2. Cytosine Arabinoside (Ara-C) and DepoCyt

IT Ara-C is another effective agent primarily used in the treatment of CNS leukemia and lymphoma for the prevention and treatment of CNS infiltration, alone or in TIT chemotherapy. As MTX IT, the dosage of Ara-C IT is calculated according to age rather than BSA [97].

Elimination of Ara-C from CSF is biphasic, with t_1/2_ of 1 h and 3.4 h. Following a single IT Ara-C dose of 30 mg, a peak CSF concentration >2 μM/L is reached and it remains above 1 μM/L during at least 24 h. Zimm et al., conducted a pharmacokinetic study in seven patients with leukemic meningitis in complete remission, showing that a single dose of 30 mg of IT Ara-C may produces CSF concentration of 1 μM/L, providing a 24-h coverage. As with intraventricular MTX, the “C × T approach” may prolong the exposure to the Ara-C. Pharmacokinetics studies have demonstrated that daily intraventricular administration of 30 mg of Ara-C for 3 consecutive days allows to maintain a cytotoxic concentration for 72 h, compared to about 24 h after a single 70 mg dose [98].

IV Ara-C is rapidly eliminated from plasma through the enzyme cytidine deaminase, which metabolize Ara-C into the inactive uracil arabinoside (Ara-U). Conversely, the conversion to Ara-U is negligible after IT administration, because of the negligible concentration of the enzyme in CSF [99]. Thus, the clearance of Ara-C from CSF is about 0.42 mL/min (eight times lower than from plasma), a value similar to the rate of CSF bulk flow (0.35 mL/min), suggesting that an irrelevant transfer rate from CSF to plasma is possible. 

After systemic administration of low dose Ara-C (such as 100 mg/mq) not significant CSF concentrations may be observed, but the use of high IV doses of Ara-C (such as >1 g/mq) produces a CSF level of Ara-C above 1 μM/L, with increased risk of unexpected neurotoxicity. The most observed neurotoxicity after Ara-C IT is a chemical aracnoiditis, other common ones are seizures, transient paraplegia, myelopathy and encephalopathy [100,101].

DTC 101 (DepoCyt) is a slow-release formulation developed to allow a prolonged exposure to cytotoxic concentration, with a lower frequency of IT Ara-C administration. Ara-C is encapsulated in multivescicular liposomes called DepoFoam, that gradually degrade, entering in the normal lipidic pathway of the body [102]. After a single dose of IT DepoCyt, the terminal t_1/2_ was increased approximately 40-fold versus conventional Ara-C (from 3.4 to about 141 h), ensuring cytotoxic concentration of about 14 days with a single dose of 50 mg of IT DepoCyt [103]. 

In a phase I trial of IT DepoCyt in children with advanced meningeal malignancies, Bomgaars et al. found a maximum-tolerated dose of 35 mg, when administered with dexamethasone with the evidence of prolonged disease stabilization or response [104]. 

The toxicity profile of DepoCyte is similar to the conventional Ara-C. Chamberlain et al. retrospectively analyzed the incidence of neurotoxicity after administration of DepoCyt in adult patients with leptomeningeal metastasis, demonstrating that DepoCyt was generally well tolerated, whereas serious treatment-related neurological complications (bacterial and chemical meningitis, hydrocephalus, conus medullaris/cauda equina syndrome, decreased visual acuity, encephalopathy, leukoencephalopathy, myelopathy, radiculopathy and seizures) occurred in about 12.5% of patients [105]. Further studies are required to assess safety and long-term toxicity of DepoCyt, especially in the pediatric population. 

#### 5.3.3. Corticosteroids

The use of hydrocortisone in combination with MTX and Ara-C in TIT chemotherapy has been showed to have an additive and synergic effect for prophylaxis and treatment of CNS leukemia and lymphoma [106,107]. 

Currently, other corticosteroids are not licensed for IT chemotherapy in children, but dexamethasone could be a potential candidate for IT administration. Non-human preclinical studies demonstrated both dexamethasone and prednisolone are quickly cleared from CSF after IT administration. Furthermore, after IV infusion dexamethasone shows higher free plasma levels compared to prednisolone, due to a lower plasma protein binding (70% for dexamethasone compared to 90% for prednisolone), resulting in greater penetration into the CSF compartment [108].

The neurotoxicity of IT corticosteroids has not been proved yet by controlled studies, although several studies have described the potential development of psychiatric adverse effects, from depressive syndromes to psychosis, caused by the use of IT corticosteroids [109].

#### 5.3.4. Thiotepa

Thiotepa is a lipid soluble alkylating agent available for IT administration, which doesn’t produce a significant advantage versus systemic thiotepa. In fact, after IV infusion both thiotepa and its active metabolite (TEPA) rapidly cross the BBB, so CSF and plasma exposure becomes almost equivalent. In addition, thiotepa and TEPA quickly diffuse out of the CSF, because of their conspicuous CSF clearance, which is nine times the rate of CSF flow [110,111].

IT thiotepa is proposed as a treatment of leptomeningeal metastases in children, but unfavorable outcomes observed in a lot of studies suggest that intrathecal thiotepa adds little to standard chemotherapy [112]. Martín Algarra et al. described two unexpected cases of severe polyneuropathy and motor loss after IT thiotepa, combined with IT MTX, IT Ara-C and radiotherapy, indicating the need for further toxicology research on IT thiotepa [113]. 

#### 5.3.5. Investigational Agents for IT Administration

Some antineoplastic agents and monoclonal antibodies have been investigated for IT administration in preclinical and phase I/II studies; still, their clinical use remains limited.

Oral 6-mercaptopurine (6-MP) is crucial for systemic maintenance therapy for ALL, but for the first time in 1991 Adamson et al. performed a phase I/II study of IT 6-mercaptopurine (6-MP) administration in pediatric patients with refractory meningeal leukemia. A 10-mg IT dose of 6-MP was administered twice weekly for 4 weeks, obtaining complete responses in four out of nine patients, with no significant toxicity [114]. 

Topotecan is a topoisomerase I inhibitor with anti-tumor activity against many adult and childhood solid tumors. In preclinical and clinical studies, the CSF penetration of the active form of topotecan was approximately 30% after systemic administration, without neurologic toxicity [115,116]. Potter et al. conducted a phase II study to evaluate the response rate and safety of IT topotecan in children with recurrent or refractory CNS leukemia or lymphoma, obtaining 38% of complete response, with mild to moderate adverse events and reversible side effects [117]. 

Busulfan is an alkylating agent usually used as an oral formulation in high-dose chemotherapy schedules for allogenic and autologous bone marrow transplantation in patients with leukemia and solid tumors. A water-soluble microcrystalline formulation of Busulfan (Spartaject Busulfan) has been found to be active in non-human neoplastic meningitis and safe following IT injection in adult patients with leptomeningeal disease [118]. In their phase I trial, Gururangan et al. established that IT Spartaject Busulfan was well tolerated in children with leptomeningeal disease from recurrent or progressive primary brain tumors, estimating 13 mg as the maximum tolerated dose [119].

Mafosfamide is a cyclophosphamide analogue which spontaneously degrades to 4-hydroxy-cyclophosphamide, a derivative which doesn’t require hepatic microsomal activation for antineoplastic activity. The effects of IT mafosfamide on various types of cancer cells were determined during preclinical investigations and clinical trials [120]. In pediatric oncology IT mafosfamide has been used safely for pediatric brain tumors with meningeal dissemination, prolonging remission of the leptomeningeal disease [121]. Lastly, in 2012 the Pediatric Brain Consortium Study performed a pilot study to investigate the feasibility of the addition of IT mafosfamide (14 mg) to a regimen of concomitant multi-agent systemic chemotherapy followed by conformal radiotherapy for children <3 years of age with newly diagnosed embryonal CNS tumors [122].

Rituximab is an anti-CD20 antibody added to chemotherapy regimens mostly for systemic B-cell lymphoma, with improved efficacy and minimal side effects. Rituximab and the other monoclonal antibodies have low penetration in CSF with heterogeneity in the distribution within the tumor. Thus, their IT employ has been limited. In their study of patients with recurrent lymphomatous meningitis, Chamberlain et al. showed that the combination of IT liposomal Ara-C and rituximab produced modest palliative activity, without additional toxicity [123].

## 6. Future Directions

In this section we show the most promising approaches currently in development for circumventing BBB and improving brain drug delivery, with the hope to forward potential future direction in this field, which may represent an example of personalized medicine.

### 6.1. Intranasal (INas) Drug Delivery 

Intranasal drug delivery is emerging as a practical, safe and non-invasive method to bypass the BBB and deliver a wide range of neurotherapeutic agents to the brain. This approach has been primarily used to investigate therapeutic possibilities for neurological diseases (such as Alzheimer′s disease, depression, migraine, schizophrenia, etc) [124,125]. 

In regard to brain tumor therapy, INas delivery has been little investigated so far, but published results indicate interesting potential for this approach in treating CNS cancer. MTX, raltitrexed and 5-fluorouracil (5-FU) have all been shown to accumulate in the brain after INas delivery [126]. After INas delivery 5-FU results in significantly greater brain exposure than IV infusion, however despite its favorable pharmacokinetic profile, this therapeutic approach should be evaluated in preclinical brain tumor models to assess efficacy [127,128]. 

### 6.2. Focused Ultrasound (FUS)—Mediated Drug Delivery 

Focused ultrasound is an emerging and non-invasive method to transiently increase permeability of the BBB [129]. In 2001, Hynynen et al. combined low power ultrasound with the delivery of IV microbubble contrast agent. When the circulating microbubbles pass through the ultrasound field, they oscillate at the frequency of the ultrasound, performing a stable cavitation [130]. Blood vessels mechanically stimulated within the stable expansion and contraction of the microbubbles, lead to transient and reproducible BBB opening, without long-term deficits in barrier function. The microbubbles concentrate the ultrasound energy, significantly reducing the amount of ultrasound pressure required to open the BBB and the risk of brain damage [131]. MRI is used for targeting the brain in order to direct drugs to the brain region of interest. This would be advantageous for chemotherapeutic treatment of brain tumors, limiting the access of antineoplastic drugs to the tumor and its periphery and avoiding the remaining healthy brain tissue [132]. 

Several preclinical studies show efficacy of the delivery of anticancer drugs into the brain after FUS induced BBB disruption, such as with doxorubicin [133,134], MTX [135], BCNU and epirubicin [136]. More investigation on the biological effects produced by FUS and its safety profile should be undertaken, still preclinical encouraging outcomes indicate its potential translation in clinical application [137]. 

### 6.3. Convection Enhanced Delivery (CED)

Convection-enhanced delivery (CED) is a technique for circumventing BBB and delivering therapeutics directly through the interstitial spaces of the CNS, using intraparenchymal microcatheters with continuous positive-pressure infusion [138]. Many clinical studies using CED of chemotherapeutic agents have been largely unsuccessful, showing which parameters should still be improved to obtain an effective clinical application [139,140]. 

Recently however Souweidane et al. reported the first trial that demonstrates the efficacy and safety of CED of a radioimmunotherapy agent targeting the glioma-associated B7-H3 antigen in children with diffuse intrinsic pontine glioma (DIPG) who have previously received radiation therapy [141]. CED could be a useful strategy to control DIPG not only in the advanced stage, but also in the initial stage, improving local disease control [142]. 

## 7. Conclusions

BBBD, IA and intracavitary chemotherapy seem to have a restricted application in the treatment of pediatric primary and metastatic CNS tumors, for their invasiveness and potentially serious adverse effects. 

Conversely, IT administration has been almost widely employed in the treatment of ALL and lymphoma to avoid the neurotoxicity of cranial irradiation. Intralumbar administration is safe and not painful if practiced with adequate deep sedation, whereas the implant of subcutaneous Ommaya reservoirs allows the release of a higher and more homogeneous drug concentration in the arachnoids space compared with intralumbar administration, avoiding stressful subsequent LP. However, Ommaya reservoirs are correlated with an elevated risk of infections or obstructive complications, which clinicians must try to prevent.

IT administration in pediatric oncology is substantially limited to MTX, Ara-C and hydrocortisone, alone or in TIT, because of the extreme neurotoxicity of many other systemic antineoplastic agents, which if accidentally given by IT are described as usually fatal. 

Moreover, the IT dose of MTX, Ara-C and hydrocortisone are age-related and not BSA-related, to reduce the incidence of neurotoxic side effects, enhancing their effectiveness, especially in younger children. 

Finally, several antineoplastic agents and monoclonal antibodies have been investigated for IT administration in preclinical and phase I/II studies, but further studies are required to improve their clinical use.

Future clinical studies should investigate the new promising approaches currently in development to improve brain drug delivery in pediatric primary and metastatic CNS tumors, such as INas or FUS-mediated drug delivery and CED.

## Figures and Tables

**Table 1:** CNS penetration of commonly used anticancer drugs (adapted from Kerr et al. [6]).

Agent	CSF: Plasma Ratio (%)
Thiotepa	>95
Carmustine	>90
Cyclophosphamide	
Total drug	50
Active metabolite	15
Cisplatinum	
Free platinum	40
Total platinum	<5
Ifosfamide	
Total drug	30
Active metabolite	15
Carboplatinum	
Free platinum	30
Total platinum	<5
6-Mercaptopurine	25
Cytarabine	15
Desametasone	15
Irinotecan	
CPT–11 lactone	14
SN–38 lactone	<8
Prednisolone	<10
Vinca alkaloids	5
Topotecan	<5
Methotrexate	3
L-asparaginase	Nd
Anthracyclines	Nd
D-Actinomycin	Nd

Nd = Not detectable.

**Table 2:** Chemical structures, relevant properties and clinical use of antineoplastic agents indicated for IT use in pediatric oncology.

Name of Drug and Structure	Chemical Formula	Properties	Indications for IT Use in Pediatric Oncology
Methotrexate (MTX) 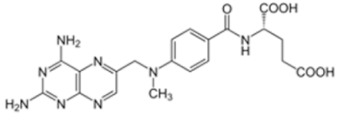	C_20_H_22_N_8_O_5_ Molecular weight: 454.45 g/mol	MTX does not penetrate the BBB in therapeutic amounts when given orally or parenterally. High CSF concentrations of the drug may be achieved by IT administration.	Acute lymphoblastic leukemia (ALL) Advanced non-Hodgkin lymphomas (NHL) CNS leukemia and lymphoma (prophylaxis)
Cytosine Arabinoside (Ara-C) 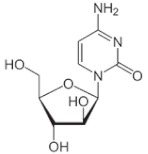	C_9_H_13_N_3_O_5_ Molecular weight: 243.22 g/mol	Only the use of high IV doses of Ara-C (>1 g/mq) produces significant CSF level of Ara-C above 1 micromol/L, with increased risk of neurotoxicity	Acute myeloid leukemia Acute lymphoblastic leukemia (ALL) CNS leukemia and lymphoma (prophylaxis)
Hydrocortisone 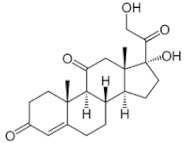	C_21_H_30_O^5^ Molecular weight: 362.47 g/mol	Good penetration into the CSF compartment after IV infusion	CNS leukemia and lymphoma (prophylaxis)
Thiotepa 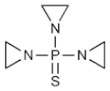	C_6_H_12_N_3_PS Molecular Weight: 189.217 g/mol	Good penetration into the CSF compartment after IV infusion	Hematopoietic stem cell transplant (HSCT) for CNS malignancy
Busulfan 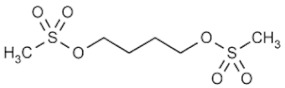	C_6_H_14_O_6_S_2_ Molecular Weight: 246.30 g/mol	Good penetration into the CSF compartment after IV infusion	Leptomeningeal disease from recurrent or progressive primary brain tumors
Topotecan 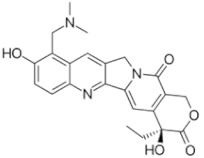	C_23_H_23_N_3_O_5_ Molecular Weight: 421.453 g/mol	Moderate penetration into the CSF (about 30%) after IV infusion	CNS leukemia or lymphoma, relapsed or refractory
6-Mercaptopurine (6-MP) 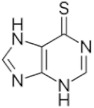	C_5_H_4_N_4_S Molecular Weight: 152.175 g/mol	Poor penetration into the CSF compartment after IV infusion	Acute lymphoblastic leukemia Lymphoblastic lymphoma
Mafosfamide 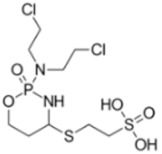	C_9_H_19_C_l2_N_2_O_5_PS_2_ Molecular Weight: 401.269 g/mol	Phase I trials showed that IV administration is unacceptable due to severe local pain at the injection site, thus mafosfamide is used through IT route	Leptomeningeal disease from recurrent or progressive primary brain tumors
Rituximab *	C_6416_H_9874_N1688O_1987_S_44_ Molecular Weight: 143859.7 g/mol	Poor penetration into the CSF compartment after IV infusion	Recurrent lymphomatous meningitis

* Rituximab is a monoclonal antibody (macromolecule) and not a small molecule like the others.

**Table 3:** Age-related dosage of TIT chemotherapy in children (adapted from Pinkel et al. [43]).

Age	Methotrexate (MTX)	Cytosine Arabinoside (Ara-C)	Hydrocortisone
≥1 year and <2 years	8	16	8
≥2 years and <3 years	10	20	10
≥3 years	12	24	12
